# In the Shadows of Motherhood: A Comprehensive Review of Postpartum Depression Screening and Intervention Practices

**DOI:** 10.7759/cureus.54245

**Published:** 2024-02-15

**Authors:** Aishwarya Gupta, Sandhya Pajai, Anusha Gupta, Aditi Singh Thakur, Shaikh Muneeba, Nitish Batra, Dharmesh J Patel

**Affiliations:** 1 Obstetrics and Gynaecology, Jawaharlal Nehru Medical College, Datta Meghe Institute of Higher Education and Research, Wardha, IND; 2 Gastroenterology, Jawaharlal Nehru Medical College, Datta Meghe Institute of Higher Education and Research, Wardha, IND; 3 Medicine, Jawaharlal Nehru Medical College, Datta Meghe Institute of Higher Education and Research, Wardha, IND

**Keywords:** mother-infant relationship, mental health support, screening tools, intervention practices, maternal mental health, postpartum depression

## Abstract

This comprehensive review delves into the multifaceted landscape of postpartum depression (PPD), exploring its prevalence, impact on maternal and infant well-being, and the efficacy of existing screening and intervention practices. PPD emerges as a critical concern, with implications extending beyond individual mental health to encompass the dynamics of mother-infant relationships and societal well-being. The analysis underscores the complexity of addressing PPD, emphasizing the challenges associated with screening tools and the importance of evidence-based interventions. A call to action resonates throughout, urging healthcare providers, policymakers, and stakeholders to prioritize mental health support for new mothers through enhanced screening protocols and improved accessibility to interventions. Furthermore, the review highlights the need for destigmatization and awareness campaigns to foster a supportive environment. Future research directions are outlined, emphasizing the refinement of screening tools, developing innovative interventions, and exploring cultural and socioeconomic influences on PPD outcomes. The review envisions a collaborative effort to dispel the shadows of PPD, striving for a future where mothers receive comprehensive support, ensuring optimal mental health and overall well-being.

## Introduction and background

Postpartum depression (PPD) is a mental health condition that affects individuals after childbirth, typically within the first few weeks to months post-delivery. Characterized by persistent feelings of sadness, anxiety, and fatigue, PPD can significantly impact a mother's ability to function and her overall well-being. Unlike the "baby blues," which are common and transient mood changes that many new mothers experience, PPD symptoms are more severe, enduring, and often require professional intervention [1].

The significance of addressing PPD extends beyond individual well-being, encompassing the well-being of both the mother and her child. PPD can interfere with the mother-infant bonding process, impede maternal caregiving abilities, and adversely affect the child's socioemotional development. Additionally, untreated PPD may lead to long-term mental health challenges for the mother, impacting her quality of life and potentially straining familial relationships. Recognizing and addressing PPD is crucial for promoting the overall health of both mothers and their infants [2].

This comprehensive review explores and analyzes various aspects of PPD screening and intervention practices. By delving into current research, established methodologies, and emerging trends, the review seeks to provide an in-depth understanding of the challenges associated with PPD identification and management. The scope encompasses screening tools, intervention strategies, cultural considerations, socioeconomic factors, and potential barriers, with the ultimate goal of informing healthcare professionals, policymakers, and researchers about practical approaches to address PPD.

## Review

Prevalence and impact of PPD

Statistics on PPD Prevalence

PPD annually affects approximately 10-15% of adult mothers, with prevalence rates exhibiting variability across countries and regions. Research indicates a range of 6.5% to 20% of women experiencing PPD. The repercussions of PPD are substantial, impacting the social and cognitive well-being of spouses, infants, and children. Identified risk factors for PPD encompass stressful life events, limited social support, a history of previous depression, and specific obstetric factors. The prevalence of PPD can be stratified based on diverse factors such as marital status, employment, infant's gender, parity, educational level, social and partner support, exposure to violence, maternal age, residence, religion, and mode of delivery [1,3,4].

Impact on Maternal Health and Child Development

PPD holds the potential to significantly impact both maternal health and the developmental trajectory of children. The effects of maternal depression during the postpartum period can extend beyond infancy, influencing toddlers, preschoolers, and even school-aged children [5]. Notably, PPD can shape the developmental milestones of school-age children. Furthermore, the mother-child relationship is susceptible to the influence of PPD, affecting crucial aspects such as bonding, breastfeeding, and the maternal role [6]. Depressed mothers may exhibit distinct patterns of attachment and caregiving, potentially resulting in developmental delays for the child [7]. Research has demonstrated an increased risk of behavioral problems and depression in later life for children born to mothers with moderate to severe PPD [8]. Recognizing the far-reaching implications, early identification and intervention for PPD emerge as imperative measures to prevent and address the adverse consequences on both maternal health and child development.

Societal Implications

PPD extends its impact beyond individual well-being, carrying significant societal implications that reverberate through the lives of women, their families, and communities. As the most prevalent complication of childbearing, PPD affects approximately 10-22% of women in their childbearing age, with onset typically occurring within the first six weeks post-delivery and potentially persisting through the first and second years postpartum [9]. The repercussions of PPD encompass adverse outcomes for both mothers and infants, manifesting as impaired bonding, developmental delays, and behavioral problems [9]. Additionally, PPD contributes to economic consequences, translating into heightened healthcare costs and diminished productivity [9]. Notably, social support plays a crucial role in mitigating the likelihood of PPD, underscoring its importance, especially for women experiencing factors like multiparity, pregnancy loss, negative body image, and those in the workforce [9]. Several studies and organizations, including the American College of Obstetricians and Gynecologists (ACOG) and the U.S. Preventive Services Task Force (USPSTF), recommend universal screening for PPD using evidence-based tools integrated into perinatal care [10]. Early identification and intervention emerge as pivotal strategies to prevent and address PPD, thereby mitigating its societal implications.

Screening methods for PPD

Overview of Screening Tools

Edinburgh postnatal depression scale (EPDS): The EPDS stands as a widely utilized screening tool aimed at identifying potential symptoms of PPD. Comprising 10 items, this questionnaire prompts respondents to reflect on their experiences over the past seven days. The cumulative score, derived by summing individual item responses, serves as an indicator, with a score of 13 or above signaling the need for follow-up care to address potential depressive symptoms. While not diagnostic, the EPDS effectively screens women who may benefit from additional support [11,12]. Demonstrating satisfactory sensitivity and specificity, this tool proves effective in identifying depressive symptoms during both the antenatal and postpartum periods. It is recommended to administer the EPDS within the first postpartum week, given the common occurrence of mood fluctuations during this period [11,13]. Completing the questionnaire is advised in consultation with a health professional, and a score of 10 points or higher warrants a conversation with a healthcare provider [14].

Beck depression inventory (BDI): The BDI is a widely recognized self-report inventory consisting of 21 multiple-choice items designed to gauge the severity of depression. As one of the most frequently employed instruments for assessing depressive symptoms, the BDI serves various purposes, including screening for depression, tracking treatment progress, and evaluating characteristic attitudes and symptoms associated with depression [15,16]. Consuming approximately 10 minutes to complete, the BDI marked a paradigm shift in healthcare professionals' understanding of depression, emphasizing the significance of the patient's thoughts in the depression experience [16]. While not a diagnostic tool, healthcare providers may use the BDI for a quick assessment. It is crucial to note that the BDI is copyrighted, and usage incurs a fee. The BDI-II represents this inventory's most commonly employed version [16], with higher total scores indicating more severe depressive symptoms.

Postpartum depression screening scale (PDSS): The PDSS emerges as a 35-item self-report instrument designed for rapid administration within 5-10 minutes. Tailored to identify high-risk individuals for PPD, the PDSS facilitates referrals for further evaluation and treatment [17]. Demonstrating robust psychometric properties, including high sensitivity and specificity for detecting major or minor PPD [18], the PDSS is exclusively crafted for postpartum women. Acknowledged by the Massachusetts Department of Public Health (MDPH) as a valuable screening tool for PPD [19], the PDSS serves as a comprehensive instrument for identifying and addressing postpartum depressive symptoms.

Evaluation of Screening Tools

Early detection and intervention for PPD are crucial components of maternal mental health care. Among the available screening tools, the EPDS is the most widely employed instrument. It is advisable to initiate screening for depressive symptoms within the first week postpartum, recognizing the prevalence of mood fluctuations during this critical period. The adoption of universal screening practices for PPD has demonstrated notable benefits, enhanced the identification and management of the condition, and ultimately improved outcomes for affected women [11,20,21]. Recognizing the significance of addressing prenatal and postpartum mood and anxiety disorders, the Postpartum Support International (PSI) advocates for universal screening employing evidence-based tools. This aligns with the updated recommendations from esteemed organizations such as the USPSTF and the ACOG, both of which emphasize the importance of incorporating depression screening into perinatal care [21,22]. These endorsements underscore the growing consensus within the healthcare community regarding the necessity of comprehensive screening efforts for PPD to ensure early identification and timely intervention, thereby prioritizing the mental well-being of new mothers.

Challenges in Screening for PPD

Screening for PPD confronts several challenges, encompassing diagnostic complexities, cultural considerations, and the imperative for appropriate follow-up and treatment. The recommendation from PSI advocates for universal screening, utilizing evidence-based tools to identify the presence of prenatal or postpartum mood and anxiety disorders. This comprehensive approach suggests collaboration with reproductive psychiatric specialists and the coordination of care among obstetric providers, midwives, and pediatricians, underscoring the multifaceted nature of addressing PPD [22]. However, healthcare professionals involved in providing mental health services to mothers with PPD face inherent difficulties in diagnosis and treatment. These challenges include the nuanced task of recognizing PPD as a legitimate condition and the knowledge gaps among healthcare providers regarding the signs and subtle symptoms of PPD [23]. The experience underscores the necessity for heightened awareness within the healthcare community regarding the nuanced nature of PPD.

Moreover, cultural factors significantly contribute to PPD screening and intervention complexity. Some mothers may be hesitant to admit symptoms due to cultural expectations, thereby influencing the recognition and subsequent treatment of PPD [24]. These cultural nuances necessitate a nuanced and culturally sensitive approach to screening and supporting affected mothers. In light of these challenges, there is a pressing need for enhanced screening practices and follow-up care to more effectively identify and support women grappling with PPD. This involves refining existing screening tools, incorporating cultural competence into healthcare practices, and improving the overall awareness and understanding of healthcare providers regarding the diverse manifestations of PPD.

Current practices in PPD intervention

Psychotherapeutic Interventions

Cognitive-behavioral therapy (CBT): CBT represents a structured and goal-oriented form of psychological treatment that has demonstrated efficacy across a spectrum of issues, including anxiety disorders, depression, eating disorders, and severe mental illnesses [25]. In this therapeutic approach, individuals are empowered to become their therapists through guided exercises during sessions and additional "homework" exercises outside the therapeutic environment. These exercises aid in developing coping skills, enabling individuals to modify their thought patterns, navigate problematic emotions, and adjust behavior accordingly [25-27]. CBT places a pronounced emphasis on current life circumstances rather than delving into the historical antecedents of difficulties [25]. Its effectiveness is notably enhanced when integrated with other treatments, such as antidepressants or alternative medications [27].

Interpersonal therapy (IPT): IPT is a time-limited, structured, and diagnosis-focused intervention designed to alleviate symptoms by improving interpersonal functioning. Thoroughly researched and successfully adapted for various psychiatric disorders, IPT typically spans 12-16 weeks with 12-20 sessions. Centered on the "here and now" of the illness, IPT addresses interpersonal challenges to help patients achieve symptom remission and enhance interpersonal functioning. Primarily utilized during the acute phase of major depression, IPT can also serve as a maintenance treatment to prevent relapse and recurrence of mood disorders like bipolar and dysthymic disorders [28-30]. IPT can be administered individually or in group formats with both nonspecific and specific elements. The therapy concentrates on four pivotal areas: relationship conflict, life changes, grief, and interpersonal role disputes. Therapists actively engage, providing non-neutral, supportive, and hopeful guidance while offering viable options for change. The patient and therapist collaboratively devise solutions to problems, with the patient implementing these solutions between sessions [30].

Pharmacological Interventions

Antidepressant medications: Antidepressants are commonly prescribed to address PPD, working to rebalance neurotransmitters in the brain that influence mood. This treatment avenue is often considered safe during breastfeeding, with minimal risk of adverse effects for infants due to the negligible transfer of these medications into breast milk, as evidenced by studies indicating no harmful impact on infants. Selective Serotonin Reuptake Inhibitors (SSRIs) typically take precedence as first-line therapy for moderate-to-severe PPD. It is crucial to collaborate closely with healthcare providers to evaluate the potential risks and benefits of specific antidepressants, particularly in the context of breastfeeding. Notably, Zurzuvae™ (zuranolone) has received approval as the initial oral medication designated for treating PPD in adults, presenting a novel option for women grappling with this condition [31-33].

Considerations and controversies: In contemplating interventions for PPD, a judicious evaluation of the benefits and risks associated with various approaches is imperative. While pharmacological interventions, notably SSRIs, are prevalent, concerns linger regarding their potential impact on breastfeeding and the developing infant [34]. A pertinent ongoing debate revolves around the balance between medication reliance and the integration of non-pharmacological interventions, such as therapy and support groups, in PPD management. Additionally, universal screening for PPD remains a contentious issue, with organizations like the ACOG and the USPSTF endorsing it. In contrast, others argue that the evidence supporting its effectiveness is inconclusive. There are concerns about potential overdiagnosis and overtreatment, emphasizing the need for a nuanced and individualized approach to PPD management. It is paramount for healthcare providers to engage in shared decision-making with their patients, considering specific circumstances and preferences to determine the most appropriate course of action [35].

Support Groups and Community-Based Interventions

Community-based interventions and support groups emerge as valuable tools in fostering mental health and overall well-being, particularly in addressing PPD. These initiatives typically entail collaborative efforts among diverse stakeholders, including community members, government agencies, and non-profit organizations [36]. An integral aspect of their success lies in actively involving community members in the planning and executing interventions, engendering a sense of ownership and empowerment. This participatory approach enhances community engagement and encourages collaboration [37]. Community interventions are pivotal in providing platforms for individuals to connect with others confronting similar challenges. This communal connection diminishes feelings of isolation and societal stigma, concurrently fostering social cohesion and resilience [37]. Furthermore, support groups within the community offer a secure and empathetic space for individuals to share their experiences openly. Participants benefit from emotional support provided by others who have navigated comparable journeys, thus creating a supportive network that aids in coping with the challenges associated with PPD [38]. Community-based interventions and support groups play a vital role in promoting mental well-being by building a sense of belonging, understanding, and shared strength within the community.

Barriers to effective PPD management

Stigma Associated With Mental Health

The stigma surrounding mental health carries substantial repercussions for individuals grappling with mental illness, manifesting in discrimination, hesitancy to seek assistance, social isolation, and diminished opportunities for work, education, or social engagement [39]. Rooted in a lack of understanding, negative attitudes, and inaccurate stereotypes about mental health conditions, stigma exacerbates mental health challenges and impedes individuals from seeking the necessary support [40]. Discrimination, the adverse judgment of someone due to their mental illness, not only worsens mental health problems but also acts as a deterrent to seeking help [41]. Stigma serves as a formidable barrier to the recovery process, fostering self-stigma wherein individuals internalize the negative perceptions associated with their mental health condition [39]. Overcoming stigma necessitates a concerted effort to challenge prejudiced beliefs, prioritizing the individual behind the mental health condition and refraining from defining them solely by their illness [41]. Recognizing the profound impact of stigma and addressing its underlying causes is instrumental in cultivating a more supportive and inclusive environment for individuals contending with mental health challenges. By fostering understanding, empathy, and a shift in societal attitudes, it becomes possible to dismantle the barriers imposed by stigma and create a space where individuals with mental illness can thrive and access the support they need.

Lack of Awareness and Education

The underrecognition and undertreatment of PPD can be largely attributed to a pervasive lack of awareness and education surrounding this condition. Many women refrain from actively seeking professional help for PPD, driven by a reluctance to disclose their feelings, which often results in delayed diagnosis and treatment [42]. The predominant focus of health professionals on the physical aspects of care during postpartum visits, coupled with the absence of a standardized U.S. protocol for identifying women at risk for PPD in the initial week after childbirth, compounds this issue [43]. Moreover, a deficiency in knowledge about PPD, coupled with concerns about not being taken seriously or facing the stigma of being labeled a 'bad mother,' acts as a significant deterrent for women in seeking the necessary help [44]. To address these barriers and ensure the timely recognition and management of PPD, there is a critical need for enhanced education and awareness among women and healthcare providers. By fostering a better understanding of PPD and its implications, as well as dismantling associated stigmas, it becomes possible to empower women to seek assistance proactively and ensure that healthcare professionals are equipped to identify and address PPD promptly.

Access to Mental Health Services

Access to mental health services in the United States is hindered by significant challenges, contributing to a pervasive mental health crisis. A comprehensive study identified insufficient access to care as a fundamental cause of the crisis, with a substantial portion of the American population lacking the necessary access to mental health services [45]. The barriers to access are multifaceted, encompassing limited options, prolonged waits for appointments, and issues related to affordability and provider availability [46]. These hurdles are particularly acute for individuals grappling with mental health challenges, who frequently confront social stigmas and encounter a scarcity of available services [47]. Compounding the issue, there exists a notable disparity between insurance coverage for mental and physical illnesses, impeding access to treatment for mental health conditions, including PPD [48]. Despite these challenges, the potential of telehealth and telemedicine services to augment access to mental health care has garnered recognition [46]. A multifaceted approach is imperative to address these barriers and enhance access to mental health services. This includes efforts to increase public awareness, improve affordability, and expand the overall availability of mental health services. By tackling these dimensions concurrently, there is a prospect of making meaningful strides in addressing the critical issue of limited access to mental health care in the United States.

Cultural and socioeconomic considerations

Cultural Differences in Perinatal Mental Health

Perinatal mental health, including PPD, is notably influenced by cultural differences. Research indicates that women from ethnic minority backgrounds may encounter barriers in accessing and engaging with perinatal mental health services, a phenomenon linked to cultural patterns such as defined roles, community support structures, and rituals [49,50]. Cultural beliefs play a crucial role in shaping the interpretation and response to PPD symptoms [50]. The social and family context, traditional practices during pregnancy and the postpartum period, and exposure to trauma and intimate partner violence are identified as risk factors for poor perinatal mental health [51]. Conversely, cultural practices can function as protective factors for women experiencing PPD [51]. To enhance perinatal mental health services, it is imperative to account for cultural and socioeconomic factors. This entails expanding opportunities for culturally appropriate education and training across various provider types [50,49]. By recognizing and incorporating cultural nuances into the design and delivery of mental health services, there is an opportunity to bridge gaps, reduce barriers, and improve the overall effectiveness of support for women from diverse cultural backgrounds during the perinatal period.

Socioeconomic Disparities in PPD Outcomes

Socioeconomic disparities in PPD outcomes are extensively documented, highlighting the unequal access to mental health treatment for women of lower socioeconomic status [52,53]. A noteworthy study revealed that women facing four socioeconomic risk factors - low income, low education, unmarried status, and unemployment - were 11 times more likely to experience PPD [53]. The ramifications of maternal depression extend to adverse economic outcomes, including unemployment and material hardship [52]. Although limited research has explored the temporal associations with the outcome, existing literature underscores the differential risks of PPD associated with factors such as race, socioeconomic status, and a history of substance abuse [52]. Screening and treating PPD are deemed cost-effective interventions, emphasizing the necessity of mandatory use of valid screening tools like the EPDS [54,55]. Additionally, the imperative of expanding opportunities for culturally appropriate education and training across various provider types is underscored for improving PPD screening and management practices [53]. By addressing these socioeconomic disparities and enhancing access to practical screening tools and culturally sensitive care, strides can be made in mitigating the impact of PPD on women of lower socioeconomic status.

Culturally Sensitive Interventions

Culturally sensitive interventions for PPD are paramount for enhancing outcomes among culturally diverse women. Healthcare providers, particularly nurses, need to be attuned to the diverse ways in which culturally varied women perceive, articulate, and disclose symptoms of PPD [56]. While some studies have explored exemplary interventions for culturally diverse women grappling with PPD, additional research is warranted to develop models for culturally competent interventions and document their outcomes [56]. Cultural factors, including the definition of roles, community support, and rituals, exert a substantial influence on the prevalence of PPD across diverse settings [56]. Recognizing the significance of cultural elements and expanding opportunities for culturally appropriate education and training across various provider types is imperative. This expansion is essential for improving the screening and management practices related to PPD [56]. Studies have indicated that cultural elements, such as the definition of roles, community support, and rituals, can elucidate the existing discrepancies in PPD prevalence [57]. Consequently, culturally sensitive interventions must be designed to consider individual differences shaped by culture, race, and socioeconomic status. This approach is crucial for preventing and addressing PPD effectively across diverse populations.

## Conclusions

In conclusion, this comprehensive review sheds light on the intricate landscape of PPD, emphasizing its profound impact on both maternal mental health and the well-being of infants. The analysis of prevalent screening tools and intervention practices reveals the complexity of addressing PPD effectively. A crucial call to action resonates throughout, urging healthcare providers, policymakers, and stakeholders to prioritize mental health support for new mothers. Enhancing routine and thorough PPD screening, coupled with improved accessibility to evidence-based interventions, stands out as a pivotal strategy. Furthermore, fostering a culture of awareness, empathy, and destigmatization surrounding mental health issues in the postpartum period is essential. Future research must delve into refining screening tools, developing innovative interventions, and exploring the nuanced influence of cultural and socioeconomic factors on PPD outcomes. Additionally, the efficacy of emerging technologies in delivering mental health support and the long-term effects of untreated PPD on maternal and child health warrant sustained investigation. In essence, this review calls for a collaborative effort to dispel the shadows of PPD, envisioning a future where mothers receive comprehensive support for optimal mental health and overall well-being.
